# Peptipedia: a user-friendly web application and a comprehensive database for peptide research supported by Machine Learning approach

**DOI:** 10.1093/database/baab055

**Published:** 2021-09-03

**Authors:** Cristofer Quiroz, Yasna Barrera Saavedra, Benjamín Armijo-Galdames, Juan Amado-Hinojosa, Álvaro Olivera-Nappa, Anamaria Sanchez-Daza, David Medina-Ortiz

**Affiliations:** Facultad de Ingeniería, Universidad Autonóma de Chile, Cinco Pte. 1670, Talca 3467987, Chile; Escuela de Ingeniería en Bioinformática, Universidad de Talca, Avenida Lircay SN, Talca 3460000, Chile; Centre for Biotechnology and Bioengineering, Universidad de Chile, Beauchef 851, Santiago 8370448, Chile; Department of Chemical Engineering, Biotechnology and Materials, Universidad de Chile, Beauchef 851, Santiago 8370448, Chile; Centre for Biotechnology and Bioengineering, Universidad de Chile, Beauchef 851, Santiago 8370448, Chile; Department of Chemical Engineering, Biotechnology and Materials, Universidad de Chile, Beauchef 851, Santiago 8370448, Chile; Centre for Biotechnology and Bioengineering, Universidad de Chile, Beauchef 851, Santiago 8370448, Chile; Department of Chemical Engineering, Biotechnology and Materials, Universidad de Chile, Beauchef 851, Santiago 8370448, Chile; Centre for Biotechnology and Bioengineering, Universidad de Chile, Beauchef 851, Santiago 8370448, Chile; Institute for Cell Dynamics and Biotechnology, Beauchef 851, Santiago 8370456, Chile; Centre for Biotechnology and Bioengineering, Universidad de Chile, Beauchef 851, Santiago 8370448, Chile; Department of Chemical Engineering, Biotechnology and Materials, Universidad de Chile, Beauchef 851, Santiago 8370448, Chile

## Abstract

Peptides have attracted attention during the last decades due to their extraordinary therapeutic properties. Different computational tools have been developed to take advantage of existing information, compiling knowledge and making available the information for common users. Nevertheless, most related tools available are not user-friendly, present redundant information, do not clearly display the data, and usually are specific for particular biological activities, not existing so far, an integrated database with consolidated information to help research peptide sequences. To solve these necessities, we developed Peptipedia, a user-friendly web application and comprehensive database to search, characterize and analyse peptide sequences. Our tool integrates the information from 30 previously reported databases with a total of 92 055 amino acid sequences, making it the biggest repository of peptides with recorded activities to date. Furthermore, we make available a variety of bioinformatics services and statistical modules to increase our tool’s usability. Moreover, we incorporated a robust assembled binary classification system to predict putative biological activities for peptide sequences. Our tools’ significant differences with other existing alternatives become a substantial contribution for developing biotechnological and bioengineering applications for peptides. Peptipedia is available for non-commercial use as an open-access software, licensed under the GNU General Public License, version GPL 3.0. The web platform is publicly available at peptipedia.cl.

**Database URL**: Both the source code and sample data sets are available in the GitHub repository https://github.com/ProteinEngineering-PESB2/peptipedia

## Introduction

Peptides play a crucial role as signalling molecules and therefore can be used for a diverse array of therapeutics, including antimicrobials, antitumourals, hormonal replacements, anti-inflammatories and anti-hypertensives (13, 14). Peptides are polymers that can be sought in natural sources or synthetically obtained; they are constituted of at least two amino acids, and their maximum length is usually about 50 amino acids (5), although there continues to be no consensus about the length delineating a peptide from a protein; therefore, sequences to 150 amino acids were considered for Peptipedia (12, 14, 18, 29).

As therapeutic agents, peptides are especially attractive because they exhibit high biological activity and specificity, alongside reduced side effects and low toxicity. Nevertheless, peptides have some disadvantages compared to other molecules. They can have relatively high synthesis costs compared to small molecule treatments or lower stability than larger proteins due of the lack of tertiary structure, making them particularly susceptible to enzymatic degradation (6, 13, 24). They can also have difficulties crossing biological membranes due to their high polarity, molecular weight and hydrophilicity (29, 32).

Despite the disadvantages mentioned, peptide researching interest has increased, resulting in a significant accumulation of new peptide sequences in conjunction with their related activities and properties. This has brought to the market over 70 peptides approved in the USA, Europe and Japan as therapeutics, more than 200 in clinical trials and more than 600 in pre-clinical tests (13, 27, 30).

One of the most significant trends in drug discovery over the last decade has been using peptides to identify new drugs or new functionalities for specific targets. In this context, computational approaches are continually developed as support tools for biological fields, where methodologies based on machine learning and data mining become relevant tools (1, 37). However, these techniques require prior knowledge, which can be obtained from biological databases that accumulate information on molecules and their characteristics. These data of interest can be collected and processed to develop a tool for solving a specific problem.

Several dedicated databases have emerged for peptide grouping, mostly, according to their activities (e.g. antimicrobial: APD3 (33), antituberculosis: AntiTBdb (31) and AntiTbPdb and antihypertensive: AHTPDB (11)) or origin source (e.g. plant: PlantPepDB (4), bacterial: BACTIBASE (7) and anuran: DADP (20)). The first web-based databases including peptides were reported in 1998 by Tossi and Sandri, 2002 (28) followed by SYFPEITHI, JenPep, FIMM and Human immunodeficiency virus (HIV) databases (2, 10, 23, 25). Then in 2003, the Antimicrobial Peptide Database (APD) appeared and has been continuously updated, but currently the link is down (33, 35), and since then, around 40 peptide databases have arisen.

Each database is useful in their specific context, but a comprehensive and integrated database focused on peptide sequences is not available so far. Besides, many of the databases present some issues which hinder their usability. Most of them do not indicate their last update, and most have not been updated since their launch, except for DRAMP, AllergenOnline, BactPepDB, DBAASP, ConoServer and APD. Other sites, including PenBase and ANTIMIC, cannot be found. Almost all databases have redundancy in their sequences (see section 1 of Supplementary Information). Others require a background in bioinformatics techniques, being unfriendly for users with no advanced computational skills. Many others do not provide a download tool: YADAMP, Quorumpeps database, DADP, BIOPEP, BioDADpep and Péptaibol; for others, the download tool is not working: PepBank, StraPep, PeptideDB, BactPepDB, MHCBN, ForPep and CancerPPD.

In this work, we present Peptipedia, a user-friendly web application and a comprehensive database to search and analyse peptide sequences, supported by machine learning applications. Peptipedia was developed to fulfil the necessities that each database cannot solve separately. We have implemented this application with a new database that encompasses the highest number of peptide sequences with reported biological activity, curated from 30 existing peptide databases. Peptipedia classifies reported biological activity for each peptide in categories and subcategories defined according to our analysis and literature (9). Our application is more than a database compilation: it is the most extensive integrated peptide persistent storage system to date. This user-friendly platform also includes a useful physicochemical and statistical properties estimator for peptides, amino acid sequences characterization, bioinformatics services and a robust binary assembled classification model support by machine learning methods for biological activity classification by a unknown peptide sequence. Both the usability and the different services enabled in our computational tool, considering the database with the highest amount of peptides with biological activity reported to date, show the significant advantages of Peptipedia and its enormous contribution to different fields of biotechnology and protein engineering.

## Methods

### Collection, pre-processing, characterization and database generation

We consolidate the information for Peptipedia by integrating the data from different computational tools and peptide sequences databases previously reported, such as APD (33), LAMP (41) and Uniprot (3), among others (see section 1 in Supplementary Information for more details). Firstly, we manually downloaded the sequences from each tool and processed them independently, generating different CSV files to facilitate their manipulation. We filtered the sequences according to their length, considering a minimum of 5 residues and a maximum of 150. Secondly, we generated a single file with all sequences, eliminating redundancy between them. For each sequence, we searched its activities, using the previous information in all databases employed to develop our web information system. It is important to note that taxonomic, structural information and specific information for particular biological activities, such as half-maximal inhibitory concentration (IC_50_) measurements and experiments, among others, were also included in Peptipedia. Furthermore, the sequences are categorized depending on whether they present modifications or non-canonical residues. Then, we used the ModLamp library (19) to characterize the peptides based on physicochemical and thermodynamic properties. Statistical properties were obtained for each sequence using the DMAKit-Lib library (16). Finally, the amino acid frequency for each sequence was obtained through scripts implemented in Python v3.6. Finally, we store the processed information in a NoSQL database, using MongoDB as a handler due to its manipulation characteristics, information extraction speed and scaling.

### Strategies for classification systems

Most sequences report a specific biological activity in terms of their biochemical roles and/or biological effects, especially in humans. We noted that a significant number of peptides are used or were designed for therapeutic purposes, but there were other seven types of peptide activity which cannot be classified as therapeutic. Consequently, we propose a classification of all peptides in eight categories, according to the information reported on all databases used to consolidate the information in Peptipedia: (i) therapeutic, (ii) immunological, (iii) cell sensing, (iv) neurological, (v) drug delivery vehicle, (vi) transit, (vii) propeptide and (viii) signal. Each category has sub-classifications within it. However, there is a small number of peptides with a variety of other activities, so we categorize them in the category (ix) ‘other activity’. All peptides with no biological activity reported are in the category (x) ‘no activity reported’. A more detailed description of each category is summarized in Table 1. Besides, the full list of categories and subcategories is available in Supplementary Figure S1 of Supplementary Information.

**Table 1.** Summary of main categories for biological activities

Biological activity	Brief description
Propeptide	Precursor with no biological activity. This molecule can be activated after a post-translational modification, such as the cleavage of a region or the addition of another molecule. Some of the molecules in this group could be in the ‘therapeutic category’ in their active form.
Signal	Used as a post-translational modification or translocation, because these peptides are useful for marking the protein secretion pathway and target location. These molecules are commonly used for the recombinant protein production, diagnosis and vaccination.
Transit	Involved in the transport of a protein encoded by a nuclear gene to a particular organelle, such as mitochondrion, chloroplast and peroxisome, among others.
Cell sensing	Activities related to cell detection mechanisms, such as quorum sensing, chemotactic movement, cell-to-cell communication and defence mechanisms, among others.
Drug delivery vehicle	Substance that helps a drug to be safely delivered to its therapeutic target, reducing toxic effects or degradation. Some examples are emulsions, polymers, semi-solid products, nanoparticles and encapsulations, among others.
Therapeutic	Able to be used for sickness treatments. The specific activity of this peptide will depend on the therapeutic target. This category includes antimicrobial, anticancer, toxic, metabolic and bioactive peptides.
Neurological	Activity related to the neurons or the nervous system. This group includes neuropeptides, brain peptides and antinociceptive activities.
Immunological	Activities related to the immune response against foreign substances. It could be related to defence mechanisms, immunomodulatory activities and wound healing, allergenic reactions and cell degranulation mechanism, among others.
Other	The activities of this group are not directly related to the other main categories. Includes four main subdivisions: mammalian and cancer cell peptides, protein peptides and surface-immobilized peptides.

One of the essential services of Peptipedia is the biological activity classification system for peptide sequences based on machine learning strategies. The training of models was based on the application of supervised learning algorithms combined with sequence encoding approaches, using physicochemical properties and digital signal processing, according to the strategies proposed by Medina-Ortiz *et al.*, 2020 (15). In this way, we generated assembled binary models to recognize activities for peptide sequences employing our categories proposed in this work. The training process was based on developing binary data sets to evaluate two categories: presence or absence of activity. Additionally, we generated each data set using the one vs rest strategy, keeping class imbalance minimum. Finally, in those models with low performance, we used the recursive binary partition strategies, according to the method proposed by Medina-Ortiz *et al.*, 2020 (17) to improve the performance of the classification assembled models.

### Implementation and Availability

Peptipedia was designed using a Model View Controller (MVC) design pattern. The view component and the controllers were implemented using JavaScript programming language through the Express framework. Display components were optimized using Bootstrap 4. All the model members, including all service disposed in this work’s proposed tool, were developed using Python v3 programming language, supported by the libraries DMAKit-Lib (16) and Scikit-Learn (21). Both the proposed software architecture and implementation features are detailed in section 2 of Supplementary Information.

## Results and Discussion

**Figure 1.**
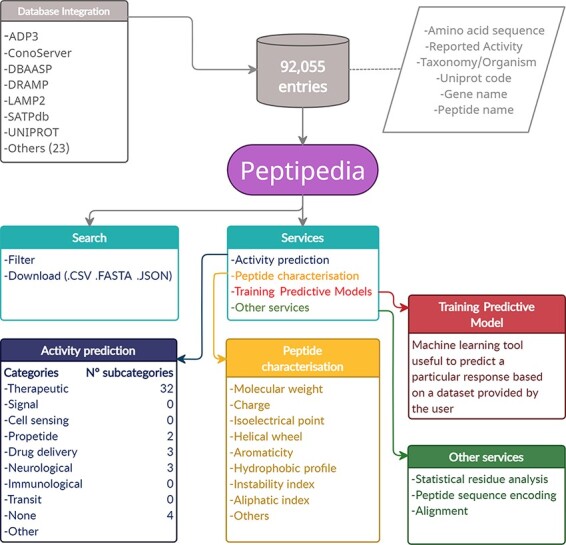
Representative scheme of building and characteristics of Peptipedia. Peptipedia is a computational tool for peptide sequence analysis. The information presented by our tool was consolidated from 30 databases, considering information on the sequence, taxonomy and different properties of stored peptides. Searching for sequences and relevant information in our web application is easy, personalized and intuitive, allowing download of the information in multiple formats. Peptipedia has enabled different tools that will help characterize and analyse sequences, as well as functionalities supported by machine learning methods that facilitate the development of predictive models and a biological activity predictor system.

Peptipedia is a user-friendly web application system to search, analyse, evaluate and characterize peptide sequences using different strategies, including machine learning and data mining techniques. This web tool has a NoSQL database system with 92 055 peptides registered and described, being the most extensive database of peptide sequences with activities reported to date. This tool reports different types of information for each sequence, considering structural, physicochemical and phylogenetic properties. Additionally, various activities previously identified for each peptide are reported and so are the databases or repositories from which they were extracted. Besides, Peptipedia has enabled the information of published patents related to previously published peptides. Finally, statistical properties related to the percentage of residues for each sequence and the average per category are included in the database, providing interesting, useful and easy-to-understand information for scientists and researchers (see Figure 1).

### Relevant tools and services available in Peptipedia

#### Searches, visualization and downloads

Different types of searches can be generated in Peptipedia, either with the sequence or through information related to its activity, physicochemical properties and frequency of residues, among other relevant information. Besides, it is possible to apply different filters to generate a personalized exploration for the user’s interest.

We develop a general summary for each search, showing statistical descriptions and various visualizations to display the information. Furthermore, we present specific details for each peptide, including thermodynamic properties, taxonomy, phylogeny, biological activity and sequence descriptors; we also show the databases where the peptide sequence was previously reported. Remarkably, Peptipedia offers specific information like IC_50_, assay information, organism evaluation and other relevant characteristics for particular activities such as antihypertensive, anti-HIV and antiviral subcategories.

Peptipedia has general and specific modules for downloading data, making it easier to obtain information and facilitate the download in CSV, FASTA and JSON formats. The complete database can even be downloaded in easily manipulatable forms that include both the sequence and its reported information.

#### Updating information

To keep the information in the persistent storage system updated, the data download service is periodically executed from different databases that make up Peptipedia, comparing the existing sequences in the current collection with the downloaded sequences, updating the information in the case that corresponds. Besides, a systematic search is done for new tools, libraries or databases reported since the last update date, inserting the appropriate records. Although it is understood that this type of maintenance is not the most suitable for a platform of this style, this type of strategy is preferred due to the curation of the information and the control of the records that are inserted into the web tool. Nevertheless, for future updates of Peptipedia, work is being done on a system for capturing new records in real time based on data mining and semantic web strategies to optimise and automate the process of updating records.

#### Services

Different services were implemented in Peptipedia to facilitate analysis and characterization of peptide sequences. We propose various services that allow characterization through physicochemical and thermodynamic properties, using the ModLamp (19) library. We also provide modules that enable the estimation of statistical properties for peptide sequences.

Bioinformatics tools such as sequence alignments are available in our web tool: using the Edlib library (26), it is possible to align any sequence against those registered in our database.

Another relevant service is the peptide biological activity classification system supported by assembled predictive models: the user can upload a list of amino acid sequences, and our tool classifies them by the categories proposed in this work, evaluating each of them. Furthermore, a peptide-encoding service is implemented using common strategies such as a One Hot Encoder and more sophisticated ones such as a Embedding through the Tape library.

Finally, Peptipedia allows the generation of predictive models for sets of peptides with specific user requirements through supervised learning algorithms and cross-validation techniques. Configuration of hyperparameters as well as coding strategy and validation method are selectable. The tool reports the performance of the generated model by the user, allowing the download to use it locally. Besides, this service enables the interpretation of the results giving different recommendations about them.

### Relevant information in Peptipedia

**Figure 2.**
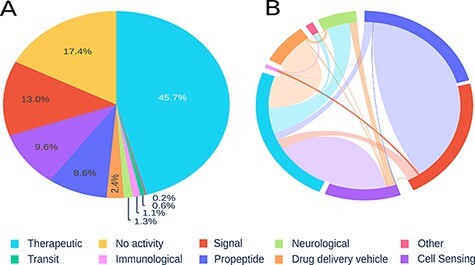
Visualization of registered peptides on Peptipedia. Representation of the information contained in Peptipedia. A: distribution of peptides according to the categories proposed in this work. B: analysis of the relationship of simultaneous activities for the same type of peptide; the most significant trends are seen between therapeutics and cell sensing peptides, and between propeptides and signal.

Using 10 previously proposed categories, we analysed the peptide sequences, identifying therapeutic peptides, signal peptides and sensory activity, as representing the highest prevalence in our records. Peptides with immunological, transit or neurological activity were least common in our database (see Figure 2A).

It is important to highlight the moonlighting characteristics of peptides. This feature is the feasibility of a peptide to present different activities at the same time (8). The mainly found tendencies of moonlighting are between the therapeutic and cell sensing peptides, and between propeptides and signal peptides. This last overlapping of activities makes sense because propeptides generally contain a signal peptide in their sequence (34), which they lose once processed (see Figure 2B). This type of property reflects the potential features of a peptide when acting as a drug or presenting different biotechnological uses. Residue frequency analysis allows evaluating amino acid trends for particular activities. We compare trends for the main reported categories, with a clear preference for arginine residues for drug delivery peptides, which can be explained because these kinds of peptides are usually designed to cross membranes, so they need a chemical affinity for negatively charged membranes, which is given by the positive charge of arginine. In contrast, signal, transit and propeptides generally show similar trends. However, no major visible patterns were identified (see Section 4 in Supplementary Information).

### The Peptipedia web interface

**Figure 3.**
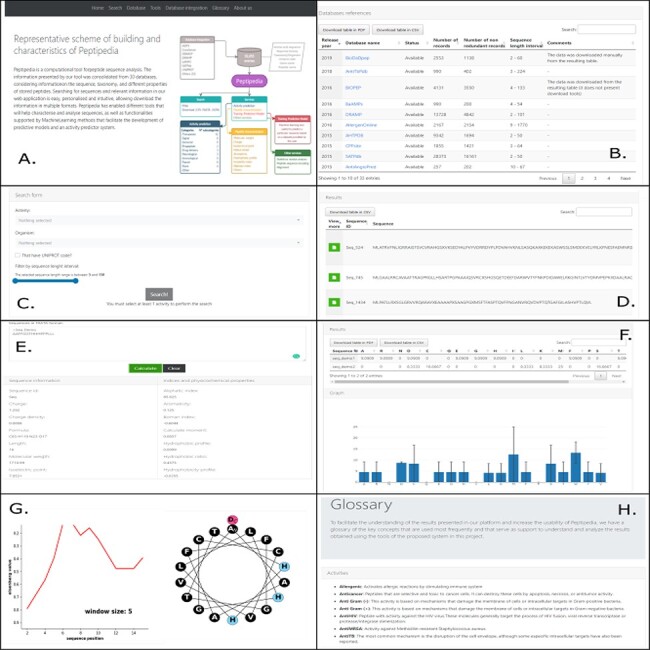
Different visualizations of the Peptipedia web interface. Home Page of Peptipedia (A). Different web tools and previously reported database used to generate the integrated collection of peptides in the proposed tool on this work (B). The search in Peptipedia is easy, you can filter by activity and add more specific filters to personalize the search. The results are disposed in a summary table (C and D). Peptipedia has different services or tools to increase the usability of the web platform; to use any tool, you need to insert a sequence in FASTA format and execute the tool. The results will be displayed depending on the selected service, which could be a summary table, graphic summary and specialized graphic (E, F and G). Finally a glossary with different terms is available on Peptipedia to help the understanding of results generated by the application tool (H).

Peptipedia presents a user-friendly web application tool to increase usability and generate a good user experience for researchers who wish to work with the computational tool proposed in this work. Figure 3 shows different visualizations available in the web interface. Upon entering the platform, a summary of the characteristics presented by Peptipedia and the availability of the information is observed (Figure 3A). Remarkably, the information extraction is made transparent, and the different links of the tools used to integrate the database collection represented by Peptipedia (3B) are enabled. The search for information in the system requires the biological activity to be analysed. Besides, it is possible to apply different filters to personalized the queries based on different user’s requirements. It is important to note that the results are shown in a summary table. It is easy to download them to be able to work on them locally (3C and 3D). The different services enabled in Peptipedia present a simple execution. They are based on entering sequences in FASTA format and generating their execution. Depending on the selected service, a table of characteristics could be displayed, as is the case of the property characterization service (3E), a summary graph of amino acid trends, in the case of the statistical characterization of sequences (3F), as well as the generation of more complex graphs such as the hydrophobicity profiles and helical wheel (3G). Finally, the tool has glossaries and messages that facilitate both the interpretation of the results and the understanding of the different concepts, properties and characteristics worked on in the application (3H).

### Binary classification of biological activities supported by assembled models

We designed and implemented 44 assembled binary classification models for biological activity of peptide sequences, considering the categories and subcategories proposed in this work. First, we encode the sequences using amino acid encoding of physicochemical properties and their representation in frequency space based on the strategies presented by Medina-Ortiz *et al.*, 2020 (15). Next, we trained predictive models based on supervised learning algorithms and assembled learning strategies (15). Besides, we employing recursive binary division strategies to optimize performance measures (17). As a validation strategy, we used *k*-fold cross-validation to avoid model overfitting. Remarkably, all the models generated presented an accuracy of over 83% (see Table 2 and section 5 of Supplementary Information for details). We previously compared the results obtained by applying this type of strategies against classical sequence coding methods, demonstrating better results (15). Furthermore, we compare our results with previously developed classification models for peptide sequences. Xiao *et al.*, 2013 (39) proposed a classification system for antimicrobial peptides with 86% accuracy; for the same task, our model achieves a performance of 88.7%. Similarly, Yi *et al.*, 2019 (40) proposed a classification system for anticancer peptides using deep learning long short-term memory model strategies, achieving an accuracy of 81.48%, while our model achieves 83.54%. Another relevant example is identifying quorum sensing peptides (QSPs): Rajput *et al.*, 2015 (22) proposed an identification system for QSPs based on sequence features in combination with support vector machine algorithms, obtaining 93% accuracy; our accuracy is slightly lower for these peptides, reaching an accuracy of 86.4%. However, we present a lower performance in particular situations than previously developed methods. Nevertheless, the proposed strategy is generic, could be applied in biological activity classification of peptide sequences problems, prediction of properties and multiple issues in protein engineering (15). Notably, we validated all our models using statistical methods. Each data set was created by selecting random samples and repeating this process 100 times, providing statistical support and demonstrating the robustness of the biological activity classification models implemented in Peptipedia.

**Table 2.** Weighted performance for binary classification models for the nine main categories proposed in this work.

Number	Category	Size data set	Weighted performance
1.	Cell sensing peptides	19 982	85.27
2.	Drug delivery	4912	86.02
3.	Therapeutic	50 000	87.32
4.	Neurological	2712	89.33
5.	Immunological	2178	86.12
6.	Other activities	490	82.98
7.	Transit peptide	1350	88.48
8.	Signal peptide	26 794	86.41
9.	Propeptide	17 768	88.63

### Case of study: How to use Peptipedia to develop predictive models

The study of anti-HIV peptides is relevant due to their potential therapeutic applications. They interact with a specific domain of the glycoprotein 41, which is their pharmacological target for inhibiting the virus fusion and entry to the host cell. Different efforts have focused on designing new sequences, either through traditional techniques such as directed evolution or rational design strategies. Both strategies currently benefit from the application of machine learning since it facilitates the simulation of the effects of new variants (36, 38).

To demonstrate the usability of different services of Peptipedia, we implemented a theoretic IC_50_ predictive model for anti-HIV peptides. First, we identify all the anti-HIV peptides using the search tool available on the web platform, with all the information provided by the downloaded tool. We then filter all sequences with a quantitative IC_50_ measure and with a defined unit of measurement, discarding the cases in which this unit was expressed using qualitative effects (low, medium or high). Finally, we prepared the data set for the training process by selecting only the peptide sequences, and the IC_50_ values were standardized so that all the records had the same unit of measurement, in this case, nM (See Table 3 for a summary of the conversions applied depending on the initial unit of measure). Based on the imposed conditions, a data set with 428 examples were generated, whose length of sequences varies between 5 and 150 residues, and the IC_50_ values are distributed between 0.01 *µ*M and 500 nM.

**Table 3.** Summary of transformations applied to standardize IC_50_ values to nM units of measure.

Number	Unity measurement actual	Change to nM
1.	mg/ml	value × 10^9^/mw
2.	ng/ml	value × 10^3^/mw
3.	*µ*M	value × 10^3^
4.	*µ*g/ml	value × 10^6^/mw

Using the generated data set and the predictive model training service enabled in Peptipedia, predictive models of IC_50_ values for anti-HIV peptide sequences were trained. We select coding by phylogenetic properties for the service configuration and apply post-processing using the alpha-structure property as a strategy for the pre-processing data set. Besides, we choose random forest as a supervised learning algorithm and we select validation strategy *k*-fold with *k* = 10.

**Figure 4.**
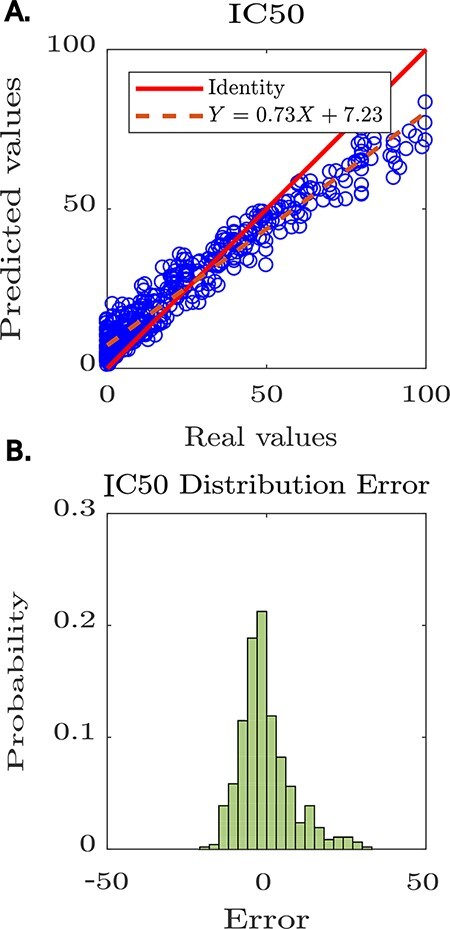
Predictive modelling of IC_50_ for anti-HIV peptides using Peptipedia. A: scatter plot prediction vs reality, denoting the performance of the predictive model. In general, there is no tendency to over-adjust or under-adjust in any particular range, which shows that the cross-validation strategies were correctly applied. B: histogram of the error distribution. The probability of error analysis indicates no tendency for significant errors that adversely alter the model predictions. The errors are mainly concentrated between -5 and 5, which is quite acceptable considering the nature of the entered values, where the largest reach 100 and the smallest are close to zero.

The tool reported the model’s performance, achieving a Pearson coefficient of 0.8 (see Figure 4A). Furthermore, Peptipedia allows us to analyse the prediction error’s randomness to determine if there are biases in the generated predictions (see Figure 4B). In this way, we demonstrated the usability of available services on Peptipedia in a specific case of study concerning developing predictive models for anti-HIV peptides. Despite the high-performance value achieved by the predictive model generated using Peptipedia, it is necessary to design more elaborate validations, compare with different coding strategies and combine with various supervised learning algorithms, explore different deep learning architectures, as well as the application of assembled learning strategies, not being the objective of this work to design and implement predictive models for the IC_50_ of anti-HIV peptide sequences.

## Conclusions

We designed and implemented Peptipedia, a web application supported by machine learning algorithms and data mining strategies to characterize and analyse peptide sequences. Additionally, our tool has the most extensive database of peptides with biological activity reported so far, with a total of 92 055 amino acid sequences integrated from 30 databases or repositories of previously reported peptides, Peptipedia has enabled different tools that will help in characterizing and obtaining statistical properties and bioinformatics analysis supported by sequence alignments, as well as services that facilitate the development of predictive models.

Additionally, the sequence and the reported biological activity information of the registered peptides are integrated into a robust binary classification system, implemented through machine learning strategies, allowing to predict putative peptide activities. Moreover, as a previous approach to wet-lab experimental work, it is possible to use these services for performing an activity screening of novel peptides with unknown biological activity. Besides, Peptipedia’s tools could improve the design of peptides since it helps to find residues patterns based on their activity.

Both the usability and the wide range of services available on Peptipedia, as well as the robustness of the predictive systems implemented, considerably improve the current state of the art, becoming an attractive alternative to existing traditional applications and a good support for research in peptide engineering and its biotechnological applications.

## Supplementary Material

baab055_Supp

## Data Availability

All codes are available at the authors’ GitHub repository https://github.com/ProteinEngineering-PESB2/peptipedia.

## References

[1] Basith S., Manavalan B., Hwan Shin T. et al. (2020) Machine intelligence in peptide therapeutics: a next-generation tool for rapid disease screening. *Med. Res. Rev.*, 40, 1276–1314.doi: 10.1002/med.21658 31922268

[2] Blythe M.J., Doytchinova I.A. and Flower D.R. (2002) Jenpep: a database of quantitative functional peptide data for immunology. *Bioinformatics*, 18, 434–439.doi: 10.1093/bioinformatics/18.3.434 11934742

[3] UniProt Consortium. (2015) Uniprot: a hub for protein information. *Nucleic Acids Res.*, 43, D204–D212.doi: 10.1093/nar/gku989 25348405 PMC4384041

[4] Das D., Jaiswal M., Khan F.N. et al. (2020) Plantpepdb: a manually curated plant peptide database. *Sci. Rep.*, 10, 1–8. 32042035 10.1038/s41598-020-59165-2 PMC7010657

[5] D’Aloisio V., Dognini P., Hutcheon G.A. et al. (2021) Peptherdia: database and structural composition analysis of approved peptide therapeutics and diagnostics. *Drug Discovery Today*, 26, 1409–1419.doi: 10.1016/j.drudis.2021.02.019 33647438

[6] Guzmán F., Barberis S. and Illanes A. (2007) Peptide synthesis: chemical or enzymatic. *Electron. J. Biotechnol.*, 10, 279–314.

[7] Hammami R., Zouhir A., Le Lay C. et al. (2010) Bactibase second release: a database and tool platform for bacteriocin characterization. *BMC Microbiol.*, 10, 1–5.doi: 10.1186/1471-2180-10-22 20105292 PMC2824694

[8] Jeffery C.J. (1999) Moonlighting proteins. *Trends Biochem. Sci.*, 24, 8–11.doi: 10.1016/S0968-0004(98)01335-8 10087914

[9] Kastin A. (2013). *Handbook of Biologically Active Peptides*. Academic Press.

[10] Korber B.T.M., Moore J.P., Brander C. et al. (1998) Hiv molecular immunology compendium. *Los Alamos National Laboratory, Theoretical Biology and Biophysics*. Los Alamos, NM.

[11] Kumar R., Chaudhary K., Sharma M. et al. (2015) Ahtpdb: a comprehensive platform for analysis and presentation of antihypertensive peptides. *Nucleic Acids Res.*, 43, D956–D962.doi: 10.1093/nar/gku1141 25392419 PMC4383949

[12] Latham P.W. (1999) Therapeutic peptides revisited. *Nat. Biotechnol.*, 17, 755–757.doi: 10.1038/11686 10429238

[13] Lau J.L. and Dunn M.K. (2018) Therapeutic peptides: historical perspectives, current development trends, and future directions. *Bioorg. Med. Chem.*, 26, 2700–2707.doi: 10.1016/j.bmc.2017.06.052 28720325

[14] Lien S. and Lowman H.B. (2003) Therapeutic peptides. *Trends Biotechnol.*, 21, 556–562.doi: 10.1016/j.tibtech.2003.10.005 14624865

[15] Medina-Ortiz D., Contreras S., Amado-Hinojosa J. et al. Combination of digital signal processing and assembled predictive models facilitates the rational design of proteins. arXiv preprint arXiv:2010.03516 (2020).

[16] Medina-Ortiz D., Contreras S., Quiroz C. et al. (2020) Dmakit: a user-friendly web platform for bringing state-of-the-art data analysis techniques to non-specific users. *Inf. Syst.*, 93, 101557.doi: 10.1016/j.is.2020.101557

[17] Medina-Ortiz D., Contreras S., Quiroz C. et al. (2020) Development of supervised learning predictive models for highly non-linear biological, biomedical, and general datasets. *Front. Mol. Biosci.*, 7, 13.doi: 10.3389/fmolb.2020.00013 PMC7031350 32118039

[18] Morrison R.T. and Boyd R.N. (1973). *Organic Chemistry*. 3rd edn. Allyn and Bacon, Boston, USA.

[19] Müller A.T., Gabernet G., Hiss J.A. et al. (2017) modlAMP: python for antimicrobial peptides. *Bioinformatics*, 33, 2753–2755.doi: 10.1093/bioinformatics/btx285 28472272

[20] Novković M., Simunić J., Bojović V. et al. (2012) Dadp: the database of anuran defense peptides. *Bioinformatics*, 28, 1406–1407. 22467909 10.1093/bioinformatics/bts141

[21] Pedregosa F., Varoquaux G., Gramfort A. et al. (2011) Scikit-learn: machine learning in python. *J. Mach. Learn. Res.*, 12, 2825–2830.

[22] Rajput A., Gupta A.K., Kumar M. et al. (2015) Prediction and analysis of quorum sensing peptides based on sequence features. *PLoS One*, 10, e0120066.doi: 10.1371/journal.pone.0120066 PMC4363368 25781990

[23] Rammensee H.G., Bachmann J., Emmerich N.P.N. et al. (1999) Syfpeithi: database for mhc ligands and peptide motifs. *Immunogenetics*, 50, 213–219.doi: 10.1007/s002510050595 10602881

[24] Rodríguez V., Asenjo J.A. and Andrews B.A. (2014) Design and implementation of a high yield production system for recombinant expression of peptides. *Microb. Cell Fact.*, 13, 1–10. 24885242 10.1186/1475-2859-13-65 PMC4022411

[25] Schönbach C., Koh J.L.Y., Sheng X. et al. (2000) Fimm, a database of functional molecular immunology. *Nucleic Acids Res.*, 28, 222–224. 10592231 10.1093/nar/28.1.222 PMC102489

[26] Sosic M. and Sikic M. (2017) Edlib: a C/C++ library for fast, exact sequence alignment using edit distance. *Bioinformatics*, 33, 1394–1395.doi: 10.1093/bioinformatics/btw753 28453688 PMC5408825

[27] Srivastava V. (ed.) (2019). *Peptide Therapeutics*, The Royal Society of Chemistry, Drug Discovery.

[28] Tossi A. and Sandri L. (2002) Molecular diversity in gene-encoded, cationic antimicrobial polypeptides. *Curr. Pharm. Des.*, 8, 743–761.doi: 10.2174/1381612023395475 11945169

[29] Uhlig T., Kyprianou T. and Martinelli F.G. et al. (2014) The emergence of peptides in the pharmaceutical business: from exploration to exploitation. *EuPA Open Proteomics*, 4, 58–69. doi: 10.1016/j.euprot.2014.05.003

[30] Usmani S.S., Bedi G. and Samuel J.S. et al. (2017) Thpdb: database of FDA-approved peptide and protein therapeutics. *PLoS One*, 12, e0181748.doi: 10.1371/journal.pone.0181748 PMC5536290 28759605

[31] Usmani S.S., Kumar R. and Kumar V. et al. (2018) Antitbpdb: a knowledgebase of anti-tubercular peptides. *Database*, 2018, bay025 doi: 10.1093/database/bay025. PMC5829563 29688365

[32] Vlieghe P., Lisowski V. and Martinez J. et al. (2010) Synthetic therapeutic peptides: science and market. *Drug Discovery Today*, 15, 40–56. doi: 10.1016/j.drudis.2009.10.009. 19879957

[33] Wang G., Li X. and Wang Z. (2016) Apd3: the antimicrobial peptide database as a tool for research and education. *Nucleic Acids Res.*, 44, D1087–D1093. doi: 10.1093/nar/gkv1278. 26602694 PMC4702905

[34] Wang J., Yin T. and Xiao X. et al. (2018) StraPep: a structure database of bioactive peptides. *Database*, 2018, bay038. doi: 10.1093/database/bay038. PMC5905355 29688386

[35] Wang Z. and Wang G. (2004) Apd: the antimicrobial peptide database. *Nucleic Acids Res.*, 32, D590–D592. doi: 10.1093/nar/gkh025. 14681488 PMC308759

[36] Wittmann B.J., Johnston K.E. and Wu Z. et al. (2021) Advances in machine learning for directed evolution. *Curr. Opin. Struct. Biol.*, 69, 11–18. doi: 10.1016/j.sbi.2021.01.008. 33647531

[37] Wu Q., Ke H. and Li D. et al. (2019) Recent progress in machine learning-based prediction of peptide activity for drug discovery. *Curr. Top. Med. Chem.*, 19, 4–16. doi: 10.2174/1568026619666190122151634. 30674262

[38] Wu Z., Johnston K.E. and Arnold F.H. et al. (2021) Protein sequence design with deep generative models. *Curr. Opin. Chem. Biol.*, 65, 18–27. doi: 10.1016/j.cbpa.2021.04.004. 34051682

[39] Xiao X., Wang P. and Lin W.-Z. et al. (2013) iamp-2l: a two-level multi-label classifier for identifying antimicrobial peptides and their functional types. *Anal. Biochem.*, 436, 168–177. doi: 10.1016/j.ab.2013.01.019. 23395824

[40] Yi H.-C., You Z.-H. and Zhou X. et al. (2019) Acp-dl: a deep learning long short-term memory model to predict anticancer peptides using high-efficiency feature representation. *Mol. Ther. Nucleic Acids*, 17, 1–9. doi: 10.1016/j.omtn.2019.04.025. 31173946 PMC6554234

[41] Zhao X., Wu H. and Lu H. et al. (2013) Lamp: a database linking antimicrobial peptides. *PLoS One*, 8, e66557. doi: 10.1371/journal.pone.0066557. PMC3688957 23825543

