# Supplementary material for: Peptipedia: a user-friendly web application and a comprehensive database for peptide research supported by Machine Learning approach
Source: Database (Oxford). 2021 Sep 3;2021:baab055. doi: 10.1093/database/baab055 (PMC8415426; doi:10.1093/database/baab055)
Supplement: baab055_Supp [file baab055_supp.zip › peptideDB_supp.pdf]

Peptipedia: a user-friendly web application and a comprehensive database for peptide research supported by Machine Learning approach

Supplementary Information

1 Developing Peptipedia Data Base

1.1 Collection of information and processing of the database

Peptipedia is a peptide database of 92,055 registers collected from 30 previously reported databases, summarized in Table 1. All the databases used to develop this tool were downloaded and preformatted independently due to they presented different formats, characteristics, and varied information, creating a single integrated repository of peptides and their properties. We eliminated the data redundancy by combining in a unique register peptides with different activities and information. Next, we remove those sequences with lengths less than two and greater than 150. We designed a NoSQL database using the document-oriented database program MongoDB. Finally, we populated the database insert all registered. Table 2 shows a summary of the component of each register in our database. All the implementation of the preformatting and evaluation of the databases was implemented in the Python v3 programming language.

Table 1: The summary databases used for collecting peptides sequences. Column # indicates all sequences and Column NR indicates non redundant sequences.

**Database**	**Access Link**	**# Seq**	**NR Seq**
APD3 [32]	http://aps.unmc.edu/AP/main.php	3167	3129
AHTPDB [14]	http://crdd.osdd.net/raghava/ahtpdb/	9342	1694
DBAASP [22]	https://dbaasp.org/home	16236	16236
CA[29]	http://www.camp.bicnirrh.res.in/	8049	7662
BACTIBASE [9]	http://bactibase.hammamilab.org/main.php	229	217
YADAMP [21]	http://yadamp.unisa.it/default.aspx	2525	2525
Quorumpeps [33]	http://quorumpeps.ugent.be/	350	350
CPPsite [1]	http://crdd.osdd.net/raghava/cppsite/	1855	1421
ArachnoServer [20]	http://www.arachnoserver.org/mainMenu.html	1838	1784
DADP [19]	http://split4.pmfst.hr/dadp/?	2571	1793

NeuroPedia [13]	http://proteomics.ucsd.edu/Software/NeuroPedia/	847	605
Erop-Moscow [34]	http://erop.inbi.ras.ru/	26697	25964
BIOPEP [17]	http://www.uwm.edu.pl/biochemia/index.php/en/biopep	4131	3550
BaAMPs [5]	http://www.baamps.it/	990	200
LAMP [35]	http://biotechlab.fudan.edu.cn/database/lamp/	22533	22279
DRAMP [11]	http://dramp.cpu-bioinfor.org	13728	4842
SATPdb [27]	https://webs.iiitd.edu.in/raghava/satpdb/	28373	16161
AllergenOnline [7]	http://www.allergenonline.org/about.shtml	2167	2154
PhytAMP [8]	http://phytamp.pfba-lab-tun.org/about.php	273	272
AntiTbPdb [30]	https://webs.iiitd.edu.in/raghava/antitbpdb/	990	402
AVPdb [24]	http://crdd.osdd.net/servers/avpdb/	2683	2359
HIPdb [23]	http://crdd.osdd.net/servers/hipdb/	981	888
Brainpeps [31]	http://brainpeps.ugent.be/	252	233
TumorHoPe [12]	http://crdd.osdd.net/raghava/tumorhope/	636	577
SPdb [3]	http://proline.bic.nus.edu.sg/spdb/	5024	4857
BioDADpep [26]	http://omicsbase.com/BioDADPep/	2553	1138
Hemolytik [6]	https://webs.iiitd.edu.in/raghava/hemolytik/	2970	1926
ConoServer [10]	http://www.conoserver.org/	7809	6685
AntiAngioPred [25]	http://clri.res.in/subramanian/tools/antiangiopred/	257	202
Uniprot [4]	https://www.uniprot.org/	12037	65

Table 2: Summary of features and values registered in PeptipediaDB

Name Feature	Description
Sequence	Peptide sequence in amino acid alphabet
Is_modify	Binary value, if 0, is non modify, else, is modify
Length	Length of sequence
In_Name-Database	Binary value, if 1, peptide is registered in $database_i$, else, is not registered in $database_i$
Activities	Binary value, if 1, peptide has the $activity_i$, else, peptide has not the $activity_i$. Full list of activity, See Figure 1.
IC_{50} information for antiviral peptides	Information related to IC_{50} measures in antiviral peptides.
IC_{50} information for Anti HIV peptides	Information related to IC_{50} measures in Anti HIV peptides.
Uniprot code	ID uniprot if peptide is registered in this database
Peptide name	Common peptide reported previously
Taxonomy	Information related to taxonomy values
Organism	Name of organism
Gene name	Name of gene
ID sequence	ID sequence registered in our database

Formula	Molecular formula of peptide sequence
Molecular Weight	Molecular weight of peptide sequence
Boman index	Boman index for peptide sequence
Charge	Charge of peptide sequence
Charge density	Charge density of peptide sequence
Isoelectric Point	Isoelectric point of peptide sequence
Instability index	Instability index for peptide sequence
Aromaticity	Aromaticity index estimated for peptide sequence
Aliphatic index	Aliphatic index for peptide sequence
Hydrophobic Ratio	Hydrophobic ratio estimated for peptide sequence
Hydrophobicity Profile	Hydrophobicity profile for peptide sequence
Hydrophobic Profile	Hydrophobic profile for peptide sequence
Momment	Momment value estimated for peptide sequence
Frequency of residues	Frequency of residues for peptide sequences expressed in percentage value.

1.2 Database Integration

Table 3 shows the number of sequences per database used in Peptipedia. Most of the sequences was obtained from the UniProt, LAMP2, SATPdb, DBAASP, DRAMP, and CAMP databases. Whereas, peptides with specific activities such as Biofilm, Neuropetids, Peptides that cross the cerebral blood barrier, among others, were collected from particular repositories to form the database with the largest number of records with activity registered and the greatest amount of information up to the moment.

Table 3: Summary of sequences used by database in Peptipedia.

Database	# Sequences	Percentage
APD	4699	5.11
AHTPDB	2719	2.95
AllergenOnline	789	0.86
AntiAngioPred	202	0.22
AntiTbPdb	388	0.42
ArachnoServer	1762	1.91
AVPdb	1906	2.07
BaAMPs	273	0.29
BACTIBASE	297	0.32
BioDaDpep	1094	1.18
BIOPEP	3383	3.67
BrainPeps	111	0.12
CAMP	8161	8.86
ConoServer	7045	7.65
CPPsite	1295	1.41

DADP	2825	3.11
DBAASP	14527	15.78
DRAMP	6562	7.13
Erop-Moscow	595	0.65
Hemolytik	2031	2.21
HIPdb	937	1.01
LAMP2	24747	26.88
NeuroPedia	662	0.72
PhytAMP	358	0.39
quorum-peps	607	0.66
SATPdb	190073	20.72
SPdb	2953	3.21
TumorHoPe	576	0.62
uniprot	21292	23.13
YADAMP	3770	4.11

It is important to note that because the sequences registered in our database may exist in different previously reported databases, the sum of the values in the # Sequences column will not add up to the total of records in Peptipedia. Similarly, this occurs for the Percentage column, where obviously the summation exceeds 100 %, which is closely related to what was previously stated.

1.3 Peptide characterization and Category classification

Once the database was generated, the sequences were classified according to activity using the previously reported databases and the set of categories proposed in this work. Then, the peptides were characterized from physicochemical properties and different components, for which scripts based on the Python v3 programming language were implemented, supported by various libraries, the most relevant being MODLAMP [18] and DMAKIT-Lib library [16].

Full visualization of all categories and subcategories proposed in this work and showing the number of peptides classified in a specific category are summarized in Figure 1. It is important to note that the subcategories' sum does not necessarily have to be equal to the peptides classified in their parent category. That is to say, Let there be two subcategories X, Y, that belong to category Z, the sum of peptides in X and Y may be different from Z. This is due to the moonlight effect of peptides, which is associated with the fact that a peptide it may have more than one activity or impact.

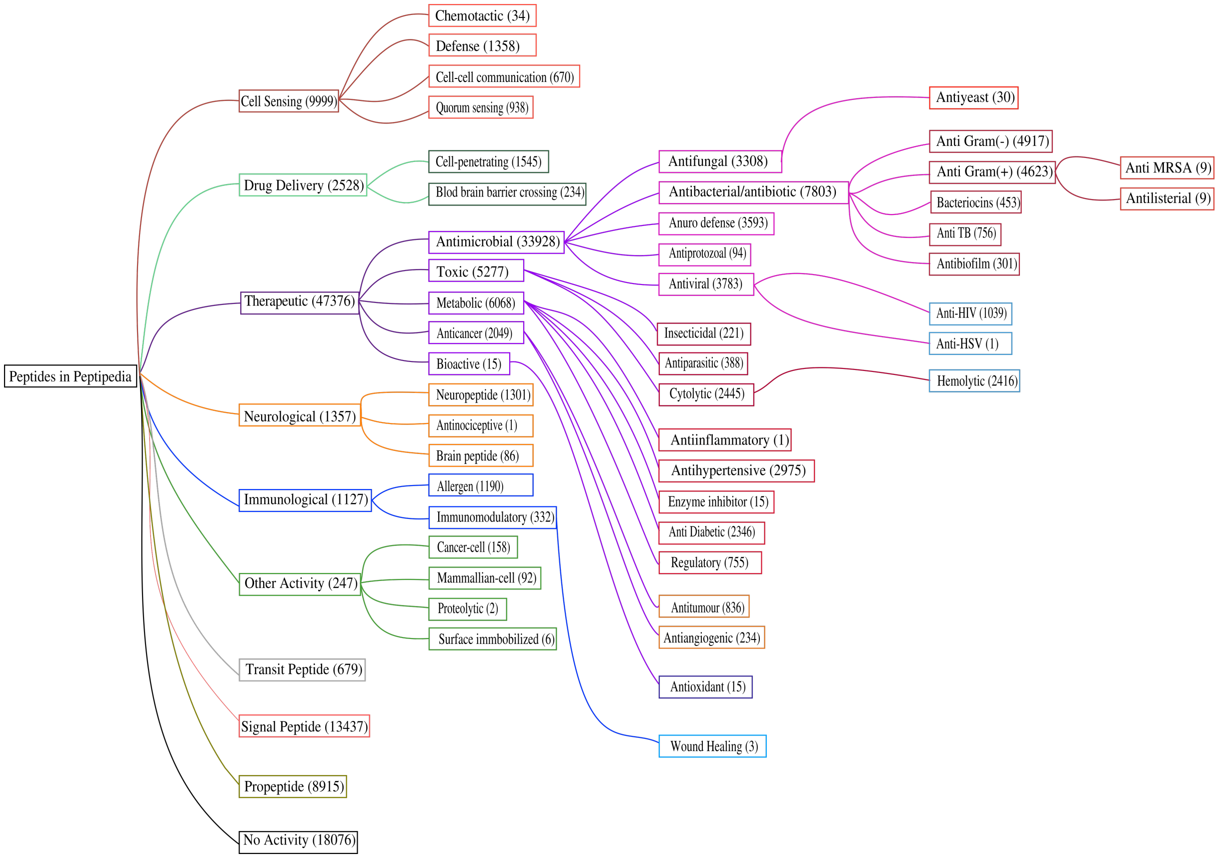

Figure 1: **Summary of categories proposed in Peptipedia.** We propose a set of categories and subcategories considering the different activities previously reported for each peptide in the databases that make up Peptipedia and rely on each activity's properties and characteristics. We present eight main categories, added to 1 category named "Other" and an additional one "No activity." The vast majority of the main categories show subcategories and so on, generating depth levels that allow understanding the various options of a peptide and its behavior at the activity level.

2 Design, Implementation, and configuration of Peptipedia

Peptipedia was designed using a Model-View-Controller (MVC) design pattern. Its software architecture is based on a client-server strategy, while all the internal logic of the system was developed under the Object-Oriented Programming paradigm (POO). Figure 2 shows the architecture proposed for the computational system developed in this work.

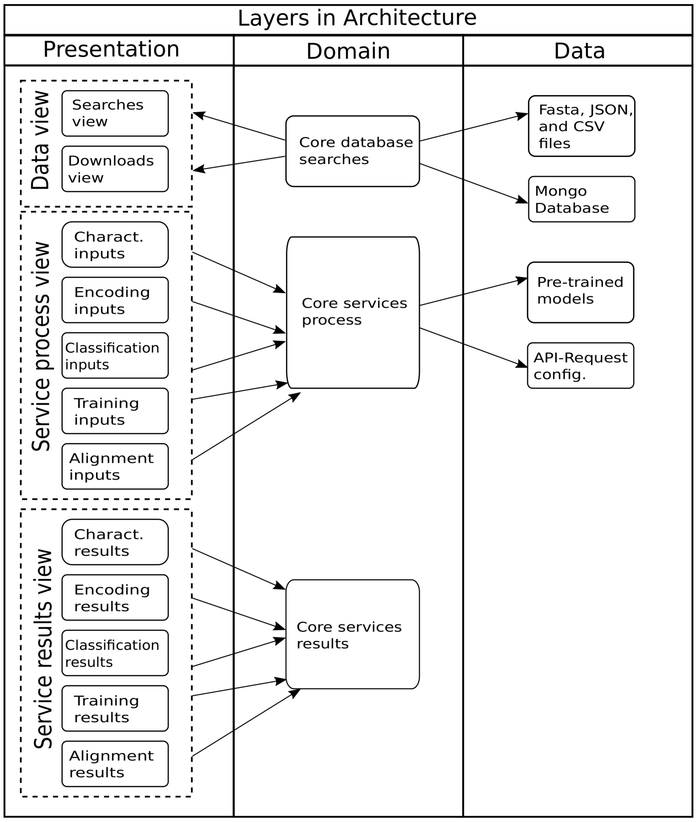

Figure 2: **Architecture design to implement Peptipedia.** A three-layer architecture consisting of Presentation, Domain, and Data was developed. The first contains all the elements associated with the view component, divided into different services or modules that make up everything. On the other hand, there is the Domain layer, which represents the controller's components and which comprise the operation of the system. Finally, the Data layer is associated with all the persistent information that is registered in the system. Besides, it also considers resources, trained predictive models, etc.

The Peptipedia implementation is divided into front-end and back-end components. For the visual element (front-end), the web technologies focused on HTML5 were used, considering HTML markup language, a CSS3 style language,

and the functionalities through JavaScript programming language. All visual orientation is optimized using the Bootstrap V4 framework. The back-end component's implementation was based on NodeJS technologies as an application server and using JavaScript programming language for all the elements implemented in Peptipedia by employing Express framework. Additionally, Python v3 programming language helped by different libraries supports the various functionalities of sequence analysis and Machine Learning implemented in the tool proposed in this work.

As a persistent storage system, a database was designed using the NoSQL MongoDB manager. Different collections are stored to contain all the information structure that supports Peptipedia and the access configurations, services, among others.

Finally, Peptipedia is hosted on a web server configured with Linux Operating System, using Debian 10 distribution. The hardware characteristics of the server are listed belong.

- 8 GB of RAM
- 4 core or vCPU
- 160 GB SSD disk
- 5 TB of traffic

Both the application and the services are encapsulated in Anaconda containers to increase the deployed application's, portability, and dependencies.

2.1 Service Implementation

Peptipedia has different services associated with the sequence's characterization using bioinformatics approaches and machine learning strategies to classify or identify peptides according to activity. Each service is encapsulated to function independently, and the results vary depending on the inputs or sequences to be analyzed and the associated configuration parameters. All services are implemented under the Python v3 programming language, and the results are generated in a JSON format to facilitate the communication between controller and view components. Remarkably, for a versed user with experience in Python, he will be able to use only the modules or library related to services available on Peptidea in the case if he does not want to use the web interface.

3 Relevant Service of Peptipedia

Peptipedia is a computational tool integrated into a NoSQL persistent storage system that records information about peptides, sequences, properties, specific characteristics, activities, and functionalities, as well as peptides' particularities in some instances. Furthermore, different tools or services are available

to interact with the database and work with peptide sequences applying bioinformatic strategies and Machine Learning and Data Mining techniques. The main components of Peptipedia are explained below, together with their relevant characteristics, functions, limitations, among others.

3.1 Search System

92055 peptides are registered in the Peptipedia database system. It is possible to view all the information in a summarized way and statistical analysis of lengths and details of activities, facilitating a general understanding of the volume of data and the data stored in Peptipedia. The system displays statistical information on the physicochemical properties of the peptides with said activity, the trend or average preference of the sequences, and different types of information of interest, as well as the use of sequences by databases, among others, considering the set of activities and sub-activities proposed in this work. Additionally, it is possible to download the information by activity, facilitating the use of the data for different data mining analyzes, applications in rational design, drug discovery, and protein engineering treatments.

Peptipedia has a search system for the information reported in the database. Different filters can be applied to do a specific search, considering physicochemical information, properties, activities, organisms, among others, allowing obtaining detailed information depending on the user's needs. All information is reported in summary form and can be downloaded in different formats (Fasta, JSON, CSV). Furthermore, each identified peptide can see its registered information in detail, displaying it merely and intuitively for correct details.

3.2 Download System

All the information available on Peptipedia can be downloaded for local use, is provided in different formats (CSV, JSON, FASTA). In general, all download systems are available automatically since they are previously developed, except for the specific queries that a user can create, made at the time of the specified request.

3.3 Tools

Different tools are available in Peptipedia, which are related to the characterization of sequences using physicochemical properties, the classification of peptide activity using pre-trained predictive models. Each of the tools, their main features, and functionalities are explained in a general way below.

3.3.1 Sequence characterization service

It is possible to estimate different physicochemical, structural, and statistical properties of the sequences concerning the percentage of residues, using a sequence or a set of amino acid sequences in FASTA format. For this, Peptipedia

takes the sequences and estimates the properties using the MODLAMP library. The sequence analysis at the statistical level is generated from specific libraries with scripts from the tool developed in work presented in this article.

3.3.2 Sequence Alignment Service

Peptipedia makes it easy to align sequences against the tool's database. First, the user must select the corresponding service and enter the sequence in Fasta format. Additionally, the tool allows selecting the sequences to align, considering categories and sequence lengths. Once the request is generated, the system collects the configuration parameters and the sequences entered and supported by the EdLib library [28] generates the sequences' alignment. As a result, the tool generates a JSON file with the aligned sequences and the similarity measure, showing the platform results in order of similarity.

3.3.3 Sequence Encoding Service

Coding sequences involves transforming categorical variables (residues) into numbers, generating numerical representations of amino acid sequences, using them in methods based on Machine Learning or data mining, either for the development of predictive models or for identifying patterns with non-bioinformatics tools. Peptipeda has implemented different coding strategies based on classic methods such as One Hot Encoder and Frequency of residues and the use of physicochemical properties and treatment through Digital signal processing. Besides, it facilitates coding through more elaborate techniques using Natural language processing (NLP) strategies, utilizing the TAPE library [2] and its different pre-trained coding models. It is essential to mention that for the encodings using physicochemical properties or Digital Signal Processing, the representations previously obtained in our previously work [15] are used. In general, Peptipedia receives sequences and removes those that do not have canonical residues, then encodes according to the selected method and generates a CSV file with the results. Besides, it is noted that the tool proposed in this work presents its zero-padding way, which is used to adapt all the encodings to the same size. Finally, the results are compressed and enabled for download by the user.

3.3.4 Classification service of peptides according to activity

Given a set of sequences, it is possible to classify them according to the categories proposed in this work, using coding methods based on physicochemical properties and representations in frequency space, followed by the use of supervised learning algorithms as Machine Learning strategies (See Section 5 for more details). Using its classification system, Peptipedia makes it easy to classify sequences in your activities. To do this, the user must select the activity/category classification service and then enter the sequences they want to work on. Once the sequences have been entered, the system collects the input data and proceeds

to encode the sequences using physicochemical properties to then apply Fourier transforms and represent the sequences in frequency space, as proposed in [15]. Then, the encoded sequences are evaluated for all the assembled classification models implemented. The response associated with whether or not it presents a particular category is obtained by using the voting system associated with the classification system. Finally, the service reports the presence or absence of all categories related to a probability percentage of the binary classification generated.

3.3.5 Training predictive models service

Given a set of sequences with their reported effects, related to the value to be predicted, it is possible to develop predictive models using Machine Learning strategies. However, this task is arduous and complicated for those users or researchers who do not have the necessary skills. Peptipedia has implemented the training service and generation of predictive models to use their data sets (peptide sequences and their corresponding effect) and train their predictive models. Once the user uploads the necessary files, he must select what type of encoding to use (the same ones enabled in the encoding service), the algorithm to use and the kind of response that will be evaluated, existing categorical (to generate classification models) or continuous (for the development of predictive models). Once the configuration is generated, the system collects the data and proceeds to train the models, using the modules used in the DMAKit-Lib [16] library. The system displays the results depending on the model-generated, allowing the visualization of a confusion matrix, evaluation of sensitivity and specificity, and a learning curve, in the case of categorical systems and the regression curve and the variability of the error, in the case of prediction systems. Additionally, for both cases, the performance and configuration hyperparameters used for the selected algorithm are shown. Remarkably, Peptipedia stands out for the usability and interpretability of the results. In this way, there are messages or information tables that support the interpretation of models and how to use them.

3.3.6 Frequency of residues analysis

It is possible to obtain the residue frequency or the amino acid content for it from the peptide sequence. Peptipedia allows obtaining these indicators through the Frequency of residues service. To do this, the user must upload a set of sequences in Fasta format and select the counting to be performed, including the residual count or the percentage estimate. The system receives the request and estimates the content according to the specifications chosen. In the case of being only a single sequence, a graph with the residue content is generated. In several sequences in the uploaded set, the system generates a table with the content for each sequence and a graph with the average range for the analyzed sequences. It is essential to clarify that all the analyzes are based on the 20 canonical residues, not considering modified peptides, non-canonical residues

such as taurine, and the sequences must only be found in the single-letter amino acid format.

4 Peptide sequence analysis

We analyze the sequences grouped by activity to identify relationships or internal patterns between the categories. To do this, we take into account all the sequences and estimate the trend of amino acid content, represented in Figure 3 for the ten main categories proposed in this work. Visual patterns highlight a difference in terms of the arginine content for the Drug Delivery category's peptides compared to the rest of the activities. Additionally, the trends for peptides from the Signal, Propeptide, and Transit categories all have similar amino acid contents, making sense among them since typically signal peptides are previously propeptides. However, more specific patterns associated with the different categories cannot be identified with this criterion, which leaves the problem open to a more methodological analysis, contemplating other characteristics and sequence representation strategies, possibly from an NLP or using frequency transformations to increase the representativeness of the sequences and their elements.

5 Developing classification models supported by Assembled strategies

We develop classification models for the categories proposed in this work. First, we discard all those categories whose number of members was less than 50. Lower numbers do not allow a straightforward generalization of the behavior or trend that describes a particular activity/category. For the development of the models, all the sequences were coded using the representations of physicochemical properties and subsequently transformed using Fourier transforms for a representation in the frequency space [15]. Then, classification models were generated using the Random Forest algorithm with hyperparameters by default, and the performance of each model was evaluated using the classic metrics. Remarkably, cross-validation techniques were applied to each training to prevent overfitting of the models. Finally, two points are relevant to consider in the proposed predictive modeling strategy. First, all data sets represent binary categories. That is, the category to be evaluated is present or absent. In this way, binary datasets are generated as input for each model. Another essential point to note is that to give statistical support to the generated classification models, the development of these one v/s rest sets was developed n times with $n = 100$ so that they report the weighted performance for each problem evaluated.

The summary of performance got for all binary classification assembled models it shows in Table 4. Remarkably, all performances are higher than 80% of accuracy. This is so relevant because, the performances reported represent the average of statistical process, demonstrating the robustness of the proposed

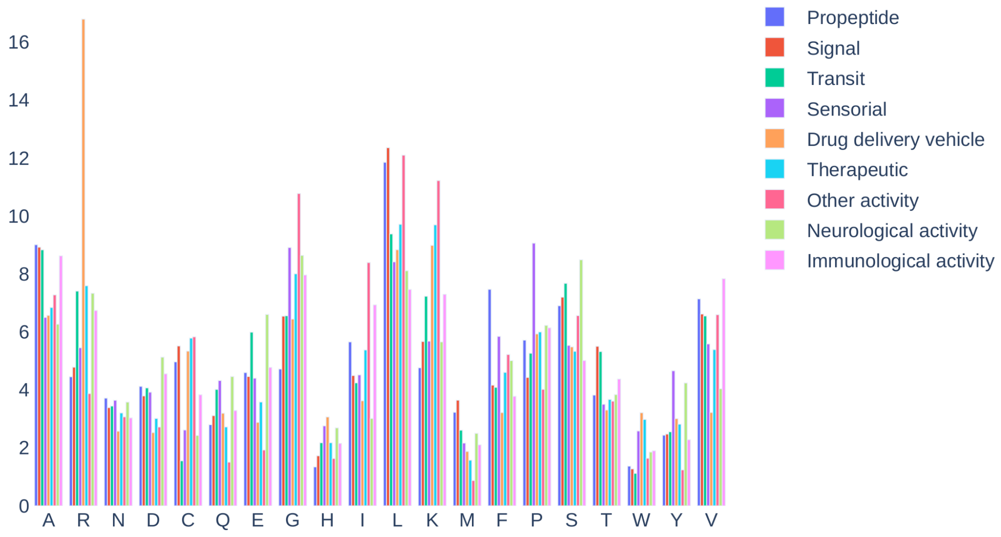

Figure 3: Average amino acid frequency of residues for peptide sequences grouped by category, using the ten main categories proposed in this work.

method and the usability of this strategies for encoding sequences in combination with assembled techniques supported by supervised learning algorithms and the different applications in protein engineering problems.

Table 4: Summary performance got by classification assembled models for all categories proposed in this work

**Activity**	**Performance**
Allergen	0.862
Anti-Angiogenic	0.830
Antibacterial-antibiotic	0.858
Antibiofilm	0.860
Anticancer	0.816
Anti-Diabetic	0.837
Antifungal	0.854
anti_gram_negative	0.871
anti_gram_positive	0.872
Anti-HIV	0.832
Antihypertensive	0.887
Antimicrobial	0.807
Antiparasitic	0.876
Antiprotozoal	0.929
Anti-TB	0.889
Antitumour	0.842
Antiviral	0.830
Anuro-defense	0.917
Bacteriocins	0.863
Blood-brain-barrier-crossing	0.787
Brain-peptide	0.876
Cancer-cell	0.826
Cell-cell-communication	0.843
Cell-penetrating	0.867
Cytolytic	0.889
Defense	0.848
Drug-delivery-vehicle	0.864
Hemolytic	0.889
Immunological-activity	0.851
Immunomodulatory	0.856
Insecticidal	0.911
Mammallian-cell	0.841
Metabolic	0.838
Neurological-activity	0.792
Neuropeptide	0.805
Other-activity	0.834
Propeptide	0.882
Quorum-sensing	0.814

Regulatory	0.863
Sensorial	0.853
Signal	0.862
Therapeutic	0.874
Toxic	0.864
Transit	0.886

References

[1] Piyush Agrawal, Sherry Bhalla, Salman Sadullah Usmani, Sandeep Singh, Kumardeep Chaudhary, Gajendra PS Raghava, and Ankur Gautam. Cpp-site 2.0: a repository of experimentally validated cell-penetrating peptides. *Nucleic acids research*, 44(D1):D1098–D1103, 2016.

[2] Tristan Bepler and Bonnie Berger. Learning protein sequence embeddings using information from structure. *arXiv preprint arXiv:1902.08661*, 2019.

[3] Khar Heng Choo, Tin Wee Tan, and Shoba Ranganathan. Spdb–a signal peptide database. *BMC bioinformatics*, 6(1):1–8, 2005.

[4] UniProt Consortium. Uniprot: a hub for protein information. *Nucleic acids research*, 43(D1):D204–D212, 2015.

[5] Mariagrazia Di Luca, Giuseppe Maccari, Giuseppantonio Maisetta, and Giovanna Batoni. Baamps: the database of biofilm-active antimicrobial peptides. *Biofouling*, 31(2):193–199, 2015.

[6] Ankur Gautam, Kumardeep Chaudhary, Sandeep Singh, Anshika Joshi, Priya Anand, Abhishek Tuknait, Deepika Mathur, Grish C Varshney, and Gajendra PS Raghava. Hemolytik: a database of experimentally determined hemolytic and non-hemolytic peptides. *Nucleic acids research*, 42(D1):D444–D449, 2014.

[7] Richard E Goodman, Motohiro Ebisawa, Fatima Ferreira, Hugh A Sampson, Ronald van Ree, Stefan Vieths, Joseph L Baumert, Barbara Bohle, Sreedevi Lalithambika, John Wise, et al. Allergenonline: a peer-reviewed, curated allergen database to assess novel food proteins for potential cross-reactivity. *Molecular nutrition & food research*, 60(5):1183–1198, 2016.

[8] Riadh Hammami, Jeannette Ben Hamida, Gerard Vergoten, and Ismail Fliss. Phytamp: a database dedicated to antimicrobial plant peptides. *Nucleic acids research*, 37(suppl_1):D963–D968, 2009.

[9] Riadh Hammami, Abdelmajid Zouhir, Christophe Le Lay, Jeannette Ben Hamida, and Ismail Fliss. Bactibase second release: a database and tool platform for bacteriocin characterization. *Bmc Microbiology*, 10(1):1–5, 2010.

[10] Quentin Kaas, Rilei Yu, Ai-Hua Jin, Sébastien Dutertre, and David J Craik. Conoserver: updated content, knowledge, and discovery tools in the conopeptide database. *Nucleic acids research*, 40(D1):D325–D330, 2012.

[11] Xinyue Kang, Fanyi Dong, Cheng Shi, Shicai Liu, Jian Sun, Jiaxin Chen, Haiqi Li, Hanmei Xu, Xingzhen Lao, and Heng Zheng. Dramp 2.0, an updated data repository of antimicrobial peptides. *Scientific data*, 6(1):1–10, 2019.

[12] Pallavi Kapoor, Harinder Singh, Ankur Gautam, Kumardeep Chaudhary, Rahul Kumar, and Gajendra PS Raghava. Tumorhope: a database of tumor homing peptides. *PLoS One*, 7(4):e35187, 2012.

[13] Yoona Kim, Steven Bark, Vivian Hook, and Nuno Bandeira. Neuropedia: neuropeptide database and spectral library. *Bioinformatics*, 27(19):2772–2773, 2011.

[14] Ravi Kumar, Kumardeep Chaudhary, Minakshi Sharma, Gandharva Nagpal, Jagat Singh Chauhan, Sandeep Singh, Ankur Gautam, and Gajendra PS Raghava. Ahtpdb: a comprehensive platform for analysis and presentation of antihypertensive peptides. *Nucleic acids research*, 43(D1):D956–D962, 2015.

[15] David Medina-Ortiz, Sebastian Contreras, Juan Amado-Hinojosa, Jorge Torres-Almonacid, Juan A Asenjo, Marcelo Navarrete, and Álvaro Olivera-Nappa. Combination of digital signal processing and assembled predictive models facilitates the rational design of proteins. *arXiv preprint arXiv:2010.03516*, 2020.

[16] David Medina-Ortiz, Sebastián Contreras, Cristofer Quiroz, Juan A Asenjo, and Álvaro Olivera-Nappa. Dmakit: A user-friendly web platform for bringing state-of-the-art data analysis techniques to non-specific users. *Information Systems*, page 101557, 2020.

[17] Piotr Minkiewicz, Jerzy Dziuba, Anna Iwaniak, Marta Dziuba, and Magorzata Darewicz. Biopep database and other programs for processing bioactive peptide sequences. *Journal of AOAC International*, 91(4):965–980, 2008.

[18] Alex T Müller, Gisela Gabernet, Jan A Hiss, and Gisbert Schneider. modlAMP: Python for antimicrobial peptides. *Bioinformatics*, 33(17):2753–2755, 05 2017.

[19] Mario Novković, Juraj Simunić, Viktor Bojović, Alessandro Tossi, and Davor Juretić. Dadp: the database of anuran defense peptides. *Bioinformatics*, 28(10):1406–1407, 2012.

[20] Sandy S Pineda, Pierre-Alain Chaumeil, Anne Kunert, Quentin Kaas, Mike WC Thang, Lien Le, Michael Nuhn, Volker Herzig, Natalie J Saez,

Ben Cristofori-Armstrong, et al. Arachnoserver 3.0: an online resource for automated discovery, analysis and annotation of spider toxins. *Bioinformatics*, 34(6):1074–1076, 2018.

[21] Stefano P Piotto, Lucia Sessa, Simona Concilio, and Pio Iannelli. Yadamp: yet another database of antimicrobial peptides. *International journal of antimicrobial agents*, 39(4):346–351, 2012.

[22] Malak Pirtskhalava, Anthony A Amstrong, Maia Grigolava, Mindia Chubinidze, Evgenia Alimbarashvili, Boris Vishnepolsky, Andrei Gabrielian, Alex Rosenthal, Darrell E Hurt, and Michael Tartakovsky. Dbaasp v3: database of antimicrobial/cytotoxic activity and structure of peptides as a resource for development of new therapeutics. *Nucleic Acids Research*, 2020.

[23] Abid Qureshi, Nishant Thakur, and Manoj Kumar. Hipdb: a database of experimentally validated hiv inhibiting peptides. *PloS one*, 8(1):e54908, 2013.

[24] Abid Qureshi, Nishant Thakur, Himani Tandon, and Manoj Kumar. Avpdb: a database of experimentally validated antiviral peptides targeting medically important viruses. *Nucleic acids research*, 42(D1):D1147–D1153, 2014.

[25] Azhagiya Singam Ettayapuram Ramaprasad, Sandeep Singh, Subramanian Venkatesan, et al. Antiangiopred: a server for prediction of anti-angiogenic peptides. *PloS one*, 10(9):e0136990, 2015.

[26] Susanta Roy and Robindra Teron. Biodadpep: A bioinformatics database for anti diabetic peptides. *Bioinformation*, 15(11):780, 2019.

[27] Sandeep Singh, Kumardeep Chaudhary, Sandeep Kumar Dhanda, Sherry Bhalla, Salman Sadullah Usmani, Ankur Gautam, Abhishek Tuknait, Piyush Agrawal, Deepika Mathur, and Gajendra PS Raghava. Satpdb: a database of structurally annotated therapeutic peptides. *Nucleic acids research*, 44(D1):D1119–D1126, 2016.

[28] Martin Sosic and Mile Sikic. Edlib: a C/C ++ library for fast, exact sequence alignment using edit distance. *Bioinformatics*, 33(9):1394–1395, 01 2017.

[29] Shaini Thomas, Shreyas Karnik, Ram Shankar Barai, Vaidyanathan K Jayaraman, and Susan Idicula-Thomas. Camp: a useful resource for research on antimicrobial peptides. *Nucleic acids research*, 38(suppl_1):D774–D780, 2010.

[30] Salman Sadullah Usmani, Rajesh Kumar, Vinod Kumar, Sandeep Singh, and Gajendra PS Raghava. Antitbpdb: a knowledgebase of anti-tubercular peptides. *Database*, 2018, 2018.

[31] Sylvia Van Dorpe, Antoon Bronselaer, Joachim Nielandt, Sofie Stalmans, Evelien Wynendaele, Kurt Audenaert, Christophe Van De Wiele, Christian Burvenich, Kathelijne Peremans, Hung Hsuchou, et al. Brainpeps: the blood–brain barrier peptide database. *Brain Structure and Function*, 217(3):687–718, 2012.

[32] Guangshun Wang, Xia Li, and Zhe Wang. Apd3: the antimicrobial peptide database as a tool for research and education. *Nucleic acids research*, 44(D1):D1087–D1093, 2016.

[33] Evelien Wynendaele, Antoon Bronselaer, Joachim Nielandt, Matthias D'Hondt, Sofie Stalmans, Nathalie Bracke, Frederick Verbeke, Christophe Van De Wiele, Guy De Tre, and Bart De Spiegeleer. Quorumpeps database: chemical space, microbial origin and functionality of quorum sensing peptides. *Nucleic acids research*, 41(D1):D655–D659, 2013.

[34] AA Zamyatnin. Erop-moscow: specialized data bank for endogenous regulatory oligopeptides. *Protein sequences & data analysis*, 4(1):49–52, 1991.

[35] Xiaowei Zhao, Hongyu Wu, Hairong Lu, Guodong Li, and Qingshan Huang. Lamp: a database linking antimicrobial peptides. *PloS one*, 8(6):e66557, 2013.
